# Crocodile Oil Disrupts Mitochondrial Homeostasis and Exacerbates Diabetic Kidney Injury in Spontaneously Diabetic Torii Rats

**DOI:** 10.3390/biom12081068

**Published:** 2022-08-02

**Authors:** Thiri Wai Linn, Anongporn Kobroob, Metas Ngernjan, Doungporn Amornlerdpison, Narissara Lailerd, Orawan Wongmekiat

**Affiliations:** 1 Nutrition and Exercise Unit, Department of Physiology, Faculty of Medicine, Chiang Mai University, Chiang Mai 50200, Thailand; thiri_wailinn@cmu.ac.th (T.W.L.); narissara.lailerd@cmu.ac.th (N.L.); 2 Division of Physiology, School of Medical Science, University of Phayao, Phayao 56000, Thailand; anongporn.ko@up.ac.th; 3 Faculty of Fisheries Technology and Aquatic Resources, Maejo University, Chiang Mai 50290, Thailand; themaetas@gmail.com (M.N.); doungporn_a@mju.ac.th (D.A.); 4 Center of Excellence in Agricultural Innovation for Graduate Entrepreneur, Maejo University, Chiang Mai 50290, Thailand; 5 Integrative Renal Research Unit, Department of Physiology, Faculty of Medicine, Chiang Mai University, Chiang Mai 50200, Thailand

**Keywords:** crocodile oil, diabetes mellitus, diabetic nephropathy, SDT rats, mitochondria, fatty acids

## Abstract

Diabetic nephropathy is currently the leading cause of end-stage renal disease (ESRD) in type 2 diabetes. Studies have suggested that supplementation with some fatty acids might reduce the risk and delay the progression to ESRD in patient with chronic kidney disease. Crocodile oil (CO) contains a variety of fatty acids, especially omega-3, -6 and -9, that have been reported to be beneficial to human health. This study examined the impact of long-term CO supplementation on the development of diabetic nephropathy in spontaneously diabetic Torii (SDT) rats. After diabetic verification, SDT rats were assigned to receive vehicle or CO at 500 and 1000 mg/kg BW, respectively, by oral gavage. Age-matched nondiabetic Sprague–Dawley rats were given vehicle or high-dose CO. After 28 weeks of intervention, CO failed to improve hyperglycemia and pancreatic histopathological changes in SDT rats. Unexpectedly, CO dose-dependently exacerbated the impairment of kidney and mitochondrial functions caused by diabetes. CO also disturbed the expressions of proteins involved in mitochondrial biogenesis, dynamics, and mitophagy. However, no significant alterations were observed in nondiabetic rats receiving high-dose CO. The findings reveal that CO has deleterious effects that aggravate diabetic kidney injury via disrupting mitochondrial homeostasis, possibly due to its improper omega-6: omega-3 ratio.

## 1. Introduction

Diabetes starts from disruption in glucose metabolism and extends to multi-organ dysfunction. The estimated global diabetes prevalence of adult populations in 2021 was 10.5% (536.6 million people) and is expected to rise to 12.2% (783.2 million) by 2045 [1]. Type 2 diabetes (T2DM) is the major form of diabetes and accounts for considerable morbidity and mortality owing to its prolonged clinical era and hyperglycemia-provoked consequences [2]. Diabetic nephropathy (DN) is a long-term complication of T2DM and the most common cause of end-stage renal disease (ESRD), which accounts for about 50% of new dialysis cases [3]. Although diabetic complications mainly originate from hyperglycemia, it involves many factors: mitochondrial dysfunction and subsequent oxidative stress generation plays a major role in the pathogenesis of various diabetes-related disorders, including DN [4].

Mitochondria are important ATP-generating organelles through oxidative phosphorylation, as well as a major source of reactive oxygen species (ROS) production [5]. They are also constantly undergoing fusion and fission to adapt intracellular energy to maintain homeostasis [5]. Mitochondria can eliminate and recycle their defective parts by a process called mitophagy [5]. The kidney is an organ with high energy consumption; thus, it is immensely sensitive to mitochondrial dysfunction [6]. Disruption in mitochondrial bioenergetics, dynamics, and oxidative phosphorylation have all been reported in diabetic kidney injury, and these changes precede the development of renal structural injury and albuminuria [6,7,8]. DN is a latent threat without symptoms in the early stages, and once the disease progresses to the condition of massive urinary protein loss, no treatment can inhibit the consequences of renal failure [8]. Therefore, strategies to retain mitochondrial homeostasis must be done early to prevent or slow the progression of diabetic kidney disease.

Nowadays, continual innovation of antidiabetic agents intends to treat diabetes and to prevent disease progression in various ways, yet drug-induced side effects have led the trend of health care to the use of dietary supplements of natural products. One of the most popular options is supplementation with monounsaturated fatty acids (MUFAs), particularly, oleic acid, and polyunsaturated fatty acids (PUFAs), namely omega-3 and omega-6 fatty acids. Oleic acid is a monounsaturated omega-9 fatty acid that is found naturally in plant products such as olive oil and in animal products such as fish oil [9,10]. Due to its cholesterol-lowering, anti-oxidative, and anti-inflammatory properties, oleic acid is associated with lower risk of CVD and beneficial effects on insulin sensitivity and β-cell survival in T2DM [11,12]. There are several experimental studies that have shown negative correlation between consumption of omega-9-rich foods and risk of T2DM [9,13]. In fact, supplementation with oleic acid reduces ROS production and, hence, protects the mitochondria from palmitic-acid-induced oxidative stress and apoptosis [14]. In addition to MUFAs, long-term consumption of dietary PUFAs could provide a considerable advantageous effect on glycemic control by increasing insulin secretion and improving insulin sensitivity [15,16]. Moreover, omega-3 PUFAs, particularly EPA and DHA, have proven cardioprotective abilities in diabetic individuals via improved lipid profiles [17] and have a protective effect against mitochondrial oxidative damage in several diseases [18].

Crocodile fat is a waste material from the leather and meat production industry. Recently, crocodile fat has been used for the extraction of crocodile oil (CO), which has been found to be rich in MUFAs and PUFAs [19]. CO has been used as an important traditional remedy in treating asthma, emphysema, cancer, burns and inflammation in Mexico, Africa, China, and several Asian countries. It has been stated that crocodile oil promotes wound healing and reduces scar formation in rats with burns [19]. Antimicrobial and antifungal activity of crocodile oil was proven in a study of Buthelezi and colleagues [20]. Although accumulating evidence indicates that MUFA-enriched fish oil or olive oil have beneficial effects in diabetes, there has been no report until now about health effects of crocodile oil in T2DM and DN. With initial efforts aimed at adding value to industrial waste by turning it into useful product, in this study we explored the effects of CO supplementation on the development and progression of diabetic renal injury using spontaneously diabetic Torii (SDT) rats. SDT rats are documented as a useful nonobese type 2 diabetic rat model for evaluating the pathogenesis and treatment of diabetic complications. They exhibit severe hyperglycemia associated with a marked decrease in insulin-secreting capacity due to tissue damage of the pancreatic islets as a major pathology of T2DM. SDT rats also develop several diabetic complications, including diabetic nephropathy, that correspond to various pathological conditions of diabetic kidney disease in humans [21,22].

## 2. Materials and Methods

### 2.1. Crocodile Oil Preparation

Fat tissues of the freshwater crocodile (*Crododylus siamensis*) were removed from the abdomens and tails of crocodiles obtained from a crocodile factory farm, and crocodile oil (CO) was extracted as described by Rattanaphot et al. (2018) [10]. In brief, fat tissues were steamed at 95 °C for 30 min, and then the resultant liquid was filtered to remove solid particles. The filtered liquid oil was subsequently centrifuged at 5000 rpm at 20 °C for 10 min. The supernatant CO was collected and sent to the Central Laboratory (Thailand) Co. Ltd. (Chiang Mai Branch, Chiang Mai, Thailand) for determination of the fatty-acid profile. The analysis was conducted by gas chromatography (Agilent 6890N, Agilent Technologies Inc., Santa Clara, CA, USA) equipped with a flame ionization detector (FID) on a capillary column (SP2560, Supelco Inc., Bellefonte, PA, USA: 100 m length, 0.25 mm internal diameter, and 0.20 µm film thickness). The column was initially set at 140 °C and held for 5 min, then continuously increased at a rate of 3 °C/min to 250 °C and held for 17 min. The various detectable peaks with different retention times were recorded. The mean value of three independent experiments was expressed as the amount of each fatty acid constituent of CO.

### 2.2. Animals and Study Protocol

The study protocol was approved by the Institutional Animal Care and Use Committee of the Faculty of Medicine, Chiang Mai University (protocol number: 27/2564) and conducted according to the Standard Operating Procedures for Animal Care and Research, Faculty of Medicine, Chiang Mai University.

Six-week-old (150–200 g) male Sprague–Dawley rats (n = 10) and spontaneously diabetic Torii (SDT) rats (n = 15) were obtained from Nomura Siam International Co., Ltd. (Bangkok, Thailand) and placed under standard temperature at 25 ± 2 °C with a 12 h light/dark cycle. Standard rat chow and pure drinking water were given ad libitum. Two weeks after acclimatization (8-weeks-old), non-fasting blood and random urine samples were collected as baseline, and samples were taken again at the age of 12 weeks for evaluation of diabetic status. At this time, oral glucose tolerance test (OGTT) was also conducted to verify diabetes development. SDT rats with impaired OGTT compared to their aged-matched nondiabetic Sprague–Dawley rats were approved as diabetes-established and were included in the study. They were then assigned into 3 groups (n = 5 each): the DMV group, which received vehicle, and the DMCO-L group and DMCO-H group, who received oral gavage with CO at a dose of 500 and 1000 mg/kg/day, respectively. The nondiabetic control Sprague–Dawley rats were randomly allotted into 2 groups (n = 5 each): the NDV group, which received vehicle, and the NDCO-H group, which was given a high dose of CO (1000 mg/kg/day). After treatment for 28 weeks (40-week-old), all rats were put in metabolic cages for 24 h urine collections. Venous blood, pancreatic tissues and both kidneys were taken under thiopental anesthesia (60 mg/kg, i.p.). One portion of kidney was immediately reserved for mitochondrial analysis, another piece was collected for light and electron microscopic studies, and all the rest were snap-frozen in liquid nitrogen then stored at −80 °C for further analyses. Pancreatic tissue was fixed in 10% formalin buffered for histological examination.

### 2.3. Oral Glucose Tolerance Test (OGTT)

OGTT was carried out at 12 weeks of age before the start of intervention. Briefly, blood samples of overnight-fasted rats were collected as baseline. D-glucose solution (2 g/kg BW) was then loading by oral gavage, and blood samples were collected at 30, 60, and 120 min after loading for determination of glucose and insulin using a commercial glucose kit (Erba Lachema, Brno, Czech Republic) and a sandwich ELISA kit for insulin (Millipore Corporation, Burlington, MA, USA). The increment of plasma glucose concentration after glucose loading was measured and expressed in terms of total area under the curve (TAUC). TAUC was calculated by adding the areas under the graph between each pair of serial observations by the trapezium rule [23].

### 2.4. Biochemical Analysis of Renal Function

Blood urea nitrogen, serum creatinine, urine creatinine, and urine microalbumin were determined by AU480 Chemistry Analyzer (Beckman Coulter Inc., Brea, CA, USA). Urinary protein was measured using a commercial assay kit (Bio-Rad Laboratories Ltd., Irvine, CA, USA). Creatinine clearance, an index of glomerular filtration rate (GFR), was computed using a standard clearance formula. Urine protein-to-creatinine ratio (UPCR) was also evaluated as an alternative test to detect proteinuria associated with diabetic kidney injury.

### 2.5. Measurement of Mitochondrial Function

#### 2.5.1. Preparation of Renal Mitochondrial Fractions

Mitochondrial fractions were prepared for further analysis as mentioned by Peerapanyasut et al. (2020) [24]. Kidney samples were homogenized in cold lysis buffer, and mitochondria were separated by differential centrifugation. The final mitochondrial pellets were dissolved in ice-cold buffer containing 5 mM KH_2_PO_4_, 250 mM sucrose, 2 mg/mL BSA, and 10 mM Tris-HCl at pH 7.2, and the protein content of the mitochondria was measured by bicinchoninic acid (BCA) assay.

#### 2.5.2. Determination of Mitochondrial Reactive Oxygen Species (ROS)

Mitochondrial ROS production was assessed using a cell-permeable fluorogenic probe 2′,7′-dichlorofluorescein diacetate (DCFDA). DCFDA can diffuse through the mitochondrial membrane and is deacetylated into membrane-impermeable non-fluorescent dichlorofluorescein (DCFH) by cellular esterases. In the presence of ROS in the mitochondria, DCFH is rapidly oxidized to highly fluorescent dichlorofluorescein (DCF). The fluorescent intensity is proportional to the amount of ROS produced by mitochondria [21]. After incubation with 2 µM DCFDA for 60 min at 25 °C, fluorescence was measured using a fluorescent microplate reader (BIOTEK^®^ Instruments Inc., Winooski, VT, USA) with excitation at 485 nm and emission at 530 nm.

#### 2.5.3. Determination of Mitochondrial Membrane Potential Change (∆Ψm)

Mitochondrial membrane potential changes (∆Ψm) were assessed by using a lipophilic cationic fluorescent dye 5,5′,6,6′-tetrachloro-1,1′,3,3′-tetraethylbenzimi-dazocarbocyanine iodide (JC-1). In physiologically polarized cells, JC-1 primarily forms red-fluorescent J-aggregates. In depolarized cells, it accumulates in mitochondria as green-fluorescent monomers. Mitochondrial suspension was incubated with 310 nM JC-I for 30 min at 37 °C. Red-fluorescent J-aggregates and monomeric forms of JC-1 were detected with excitation/emission wavelengths of 485/590 nm and 485/530 nm, respectively, using a fluorescent microplate reader (BIOTEK^®^ Instruments Inc., Winooski, VT, USA). The changes in ∆Ψm were assessed by red/green ratio, with a decrease in the ratio indicating mitochondrial depolarization.

### 2.6. Light Microscopic Studies

Tissues samples were fixed in 10% neutral formaldehyde, dehydrated in graded alcohol, embedded in paraffin wax, and then sectioned. After deparaffinizing and staining with periodic acid–Schiff (PAS) and hematoxylin and eosin (H&E), 4 µm-thick sections were examined under a Leica DM750 photomicroscope (Leica Microsystems, Heerbrugg, Switzerland). Up to ten randomly selected separate nonoverlapping microscopic fields for each kidney section were examined under light microscope at 400× magnification. H&E staining was used to evaluate endothelial proliferation, mesangial proliferation, matrix accumulation, and interstitial inflammation, and PAS staining was examined for interstitial fibrosis. The severity of injury was graded (3 rats/group) by an experienced pathologist who was unaware of the treatment groups using a modified method previously described [25,26]. Arbitrary scores (au) were applied as follows: no change (0), no change or histopathological changes <10%; mild (0.5), 10–25%; moderate (1.0), 25–50%; and severe (1.5), >50%. Then, a mean score was calculated.

### 2.7. Electron Microscopic Studies

Transmission electron microscopy was used to examine kidney ultrastructure by applying the protocol described by Peerapanyasut and colleagues (2020) [24]. Renal cortical sections 60–80 nm thick were stained with uranyl acetate and lead citrate, then studied under a JEM-2200 FS transmission electron microscope (JEOL, Tokyo, Japan). Analysis of mitochondrial morphology was performed according to the method previously described [7]. Aperio Imagescope Version 12.3.2.5030 (Informer Technologies, Inc., Los Angeles, CA, USA) was used to measure mitochondrial length and width and to count the number of mitochondria. The aspect ratio (length/width) was also calculated.

### 2.8. Western Blot Analysis

The kidney tissue samples were homogenized in ice-cold lysis buffer with 1% protease inhibitor. Total protein concentration was determined with a Bradford protein assay kit (Bio-Rad Laboratories Ltd., Irvine, CA, USA) using bovine serum albumin (BSA) as standard. The proteins were electrophoresed by 10% SDS-polyacrylamide gel electrophoresis (SDS-PAGE), subsequently blotted to a nitrocellulose membrane, and blocked for 1 h at room temperature with 5% BSA in 0.1% Tris-buffered saline-Tween 20 (TBST). The membrane was then probed with primary antibodies against AMP-activated protein kinase (AMPK), phospho-AMPK at Thr172 (p-AMPK^Thr172^), Peroxisome proliferator-activated receptor gamma coactivator 1 alpha (PGC-1α), Glyceraldehyde 3-phosphate dehydrogenase (GAPDH) as a loading control (Millipore Corporation, Burlington, MA, USA), sirtuin 3 (SIRT3), mitofusin 2 (MFN2), phospho-dynamin-like protein 1 at Ser616 (p-DRP1^Ser616^), PTEN-induced putative kinase 1 (PINK1), and PARKIN (Cell Signaling Technology, Danvers, MA, USA) overnight at 4 °C, followed by incubation with specific horseradish peroxidase-conjugated secondary antibodies (Abcam Inc., Waltham, MA, USA). The protein bands were visualized with an enhanced chemiluminescence (ECL) detection reagent using a ChemiDoc™ Touch Imaging System (Bio-Rad Laboratories Ltd., Irvine, CA, USA). The protein intensity was quantified using the Image J program (National Institute of Health, Bethesda, MD, USA).

### 2.9. Statistical Analysis

All values are stated as mean ± SEM. Multiple comparisons between experimental groups were calculated using one-way analysis of variance (ANOVA) followed by post-hoc Fisher’s least-significant difference test. Standard *t*-test was used for comparison between the two groups (as appropriate). Statistical analysis was conducted using SPSS for Windows version 25.0 (IBM Corporation, Armonk, NY, USA). Values of *p* less than 0.05 were considered significant differences between groups.

## 3. Results

### 3.1. Gas Chromatography Reveals Omega 9 Oleic Acid as a Major Constituent of CO

As shown in Table 1, CO consisted of both saturated fatty acids (SFAs) and unsaturated fatty acids (MUFAs and PUFAs). Of these, MUFAs accounted for nearly 50% of total fatty acids in CO, with oleic acid, one of the omega-9 fatty acids, the most-abundant MUFA. CO also contained similar amounts of SFAs and PUFAs, with palmitic acid the main SFA, and omega-3 (ALA, EPA, and DHA) and omega-6 (LA) fatty acids constituting the remainder of the PUFAs.

### 3.2. SDT Rats Proceed to Pre-Diabetic Stage at the Age of 12 Weeks

Bodyweight, food intake, non-fasting plasma glucose, serum creatinine, and urine protein-to-creatinine ratio (UPCR) were very similar between nondiabetic (ND) and SDT rats at the age of 8 weeks (Table 2). Four weeks later, it was found that these parameters in SDT rats remained comparable to their aged-matched ND controls. To determine whether the SDT rats progressed to diabetes during this time, an oral glucose tolerance test (OGTT) was performed. It was noted that, while fasting plasma glucoses were not significantly different between the two groups, the levels of fasting plasma insulin and the total area under the curve (TAUC) of glucose was markedly and significantly higher in the SDT rats compared to the ND rats. These results showed that SDT rats had impaired glucose tolerance and compensated by increasing insulin production, suggesting that the SDT rats at this age (12 weeks) had advanced to the pre-diabetic stage. Most importantly, there was no evidence of renal impairment at this age, since the levels of serum creatinine and UPCR remained unaltered. Thus, we started the intervention with CO at this point to inspect the role of CO in diabetic renal complications.

### 3.3. CO Does Not Attenuate Diabetes-Induced Metabolic Impairments and Abnormal Pancreatic Islet Histopathology

Table 3 shows metabolic parameters in all studied groups at 40 weeks of age after intervention with CO for 28 weeks. All SDT rats in DMV, DMCO-L, and DMCO-H groups exhibited significant hyperglycemia and hypoinsulinemia. This was associated with failure to gain weight despite a marked increase in food intake compared to the nondiabetic (NDV and NDCO-H) groups. These observations are consistent with the hallmarks of T2DM, suggesting that all SDT rats in our study fully developed T2DM at this age. Long-term CO supplementation for the SDT rats (DMCO-L and DMCO-H), regardless of dose, had no effect on modifying any of these metabolic impairments caused by diabetes. Administration of high doses of CO to nondiabetic rats (NDCO-H) also showed no significant changes to any of the metabolic parameters examined from the vehicle-treated nondiabetic (NDV) group.

**Figure 1:**
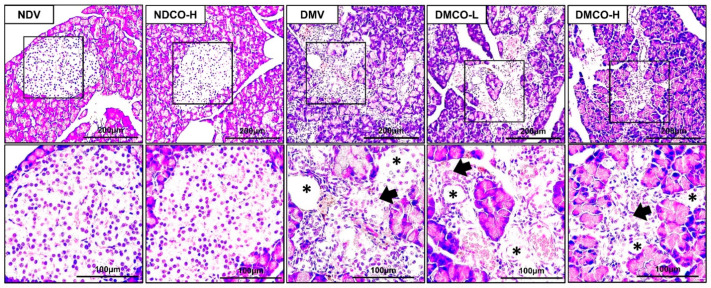
Effects of crocodile oil on histological appearance of pancreatic islets of Langerhans (H&E staining, 10× upper row and 40× lower row). Apoptotic cells (arrow) and vacuolization (asterisk) are observed. NDV: nondiabetic treated with vehicle; NDCO-H: nondiabetic treated with high-dose crocodile oil; DMV: diabetic treated with vehicle; DMCO-L: diabetic treated with low-dose crocodile oil; and DMCO-H: diabetic treated with high-dose crocodile oil.

Consistent with metabolic outcomes, CO supplementation for the nondiabetic rats did not disturb normal pancreas histology (Figure 1). Well-demarcated round or elongated pancreatic islets were found all over the pancreas. Within the islets, normal, healthy β-cells were observed in the sections from both the NDV and NDCO-H groups.

However, in pancreatic sections taken from diabetic rats, abnormal appearances such as disorganized cells with developed degranulation, apoptotic cells, vacuolization, congestion, and infiltration of leucocytes were observed. These abnormalities were very similar among all diabetics whether or not they were supplemented with CO.

### 3.4. CO Supplementation Aggravates Renal Functional and Structural Damage in Diabetic Kidneys

**Figure 2:**
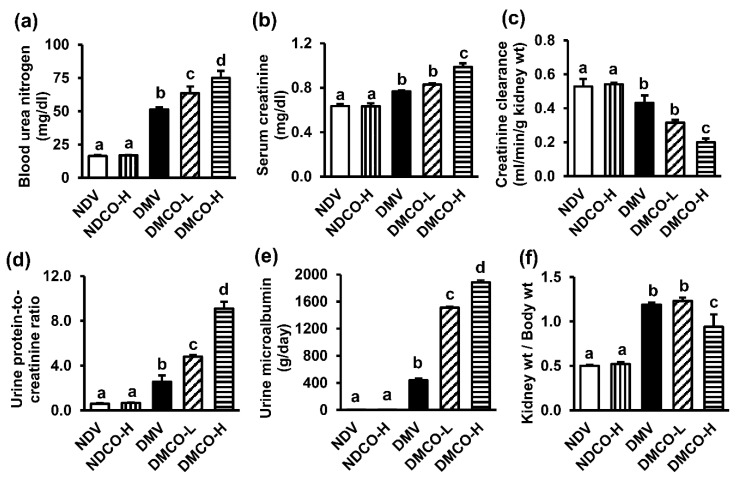
Effects of crocodile oil on renal functional parameters at the age of 40 weeks: (**a**) blood urea nitrogen; (**b**) serum creatinine; (**c**) creatinine clearance; (**d**) urine protein-to-creatinine ratio; (**e**) urine microalbumin; (**f**) kidney-to-bodyweight ratio. Values are mean ± SEM (n = 5 each). NDV: nondiabetic treated with vehicle; NDCO-H: nondiabetic treated with high-dose crocodile oil; DMV: diabetic treated with vehicle; DMCO-L: diabetic treated with low-dose crocodile oil; and DMCO-H: diabetic treated with high-dose crocodile oil. Different lowercase letters denote statistical differences at *p* < 0.05 between groups.

The diabetic control group (DMV) showed significant increases in blood urea nitrogen, serum creatinine, urine protein-to-creatinine ratio, and urine microalbumin, as well as decreased creatinine clearance (Figure 2a–e) compared with the NDV group, indicating that the SDT rats had developed diabetic nephropathy by this time. Interestingly, diabetes-induced renal dysfunction was significantly worse in a dose-dependent fashion in diabetic groups treated with CO, whereas no change in renal function was observed after CO supplement in the NDCO-H group.

**Figure 3:**
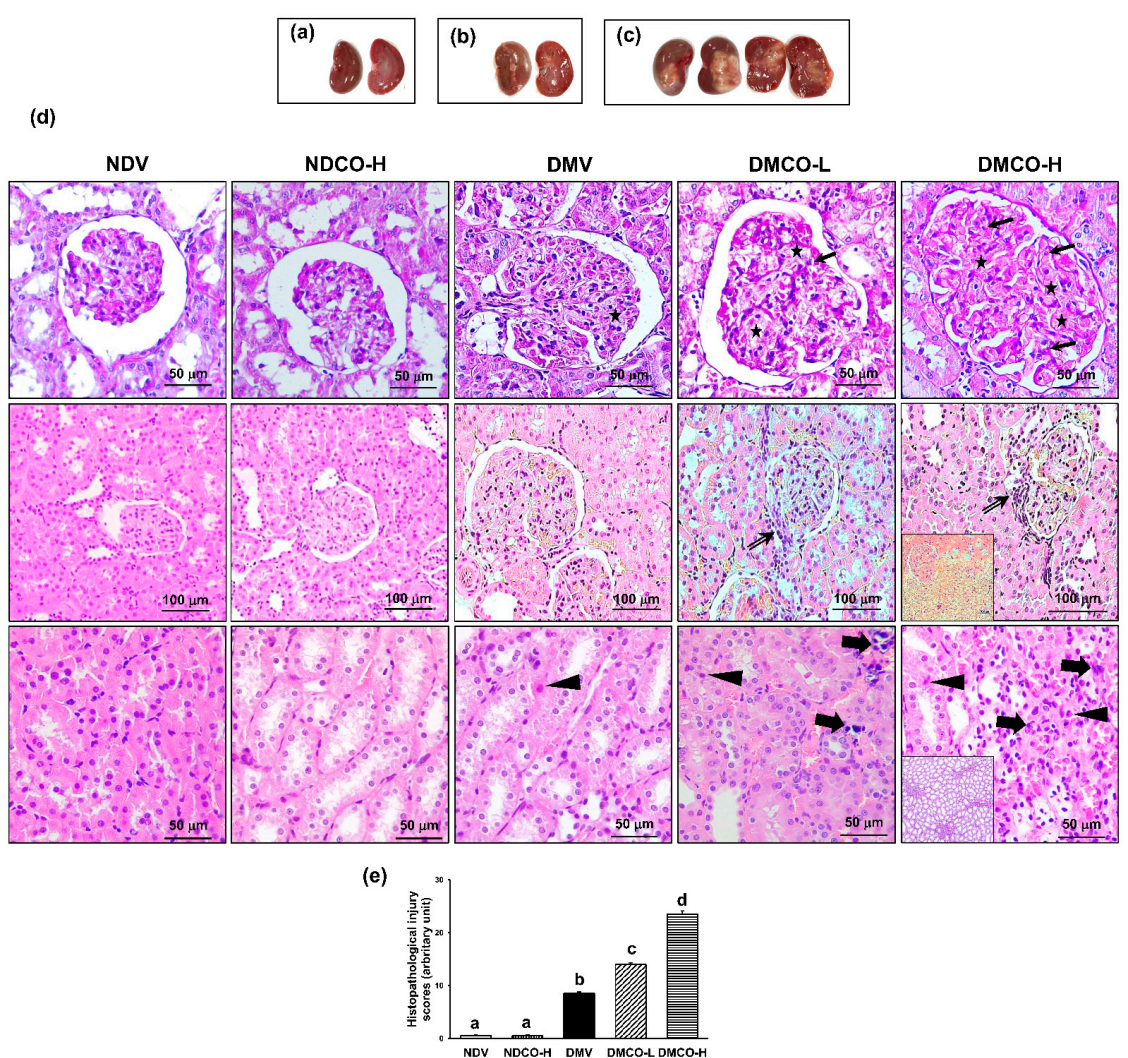
Effects of crocodile oil on renal structural changes at the age of 40 weeks: (**a**–**c**) gross findings of kidneys with cross-sectional view; (**d**) histological alterations with PAS (upper row, 40×) and H&E staining (glomeruli in middle row, 10×, and renal tubules in lower row, 40×) showing endothelial cell proliferation (arrow), matrix accumulation (star), perihilar mesangial cell proliferation (double arrow), apoptotic cell (triangle), inflammatory cell infiltration (block arrow), appearance of abnormal cells (inset, H&E staining), and interstitial fibrosis (inset, PAS staining) within DMCO-H. (**e**) Histopathological injury score: values are mean ± SEM (n = 3 each). NDV: nondiabetic treated with vehicle; NDCO-H: nondiabetic treated with high-dose crocodile oil; DMV: diabetic treated with vehicle; DMCO-L: diabetic treated with low-dose crocodile oil; and DMCO-H: diabetic treated with high-dose crocodile oil. Different lowercase letters denote statistical differences at *p* < 0.05 between groups.

The kidney-weight-to-bodyweight ratio was equivalent between the nondiabetic groups (Figure 2f), whereas it was significantly higher in all diabetes models irrespective of CO supplementation. In views of gross appearance, kidneys taken from NDV, NDCO-H, and DMV groups (Figure 3a) showed normal color and characteristics. Surprisingly, the kidneys of CO-treated diabetic rats (Figure 3b,c) showed abnormal tissue growth, especially in the DMCO-H group (Figure 3c), where a mass occupied almost the entire width of the kidney.

At the microscopic level (Figure 3d), both the NDV and NDCO-H groups showed apparently normal glomeruli and tubules, whereas the DMV group demonstrated glomerular endothelial cell proliferation, matrix accumulation, mesangial cell proliferation, and interstitial inflammation. These findings were also present in the DMCO-L and DMCO-H groups, together with perihilar mesangial cell proliferation, interstitial fibrosis, and tubular cell apoptosis with severity increasing in a dose-dependent manner (Figure 3e). The results suggest that diabetic kidney injury was augmented by long-term CO supplementation.

### 3.5. CO Worsens Mitochondrial Dysfunction and Ultrastructural Abnormalities in Diabetic Kidneys

**Figure 4:**
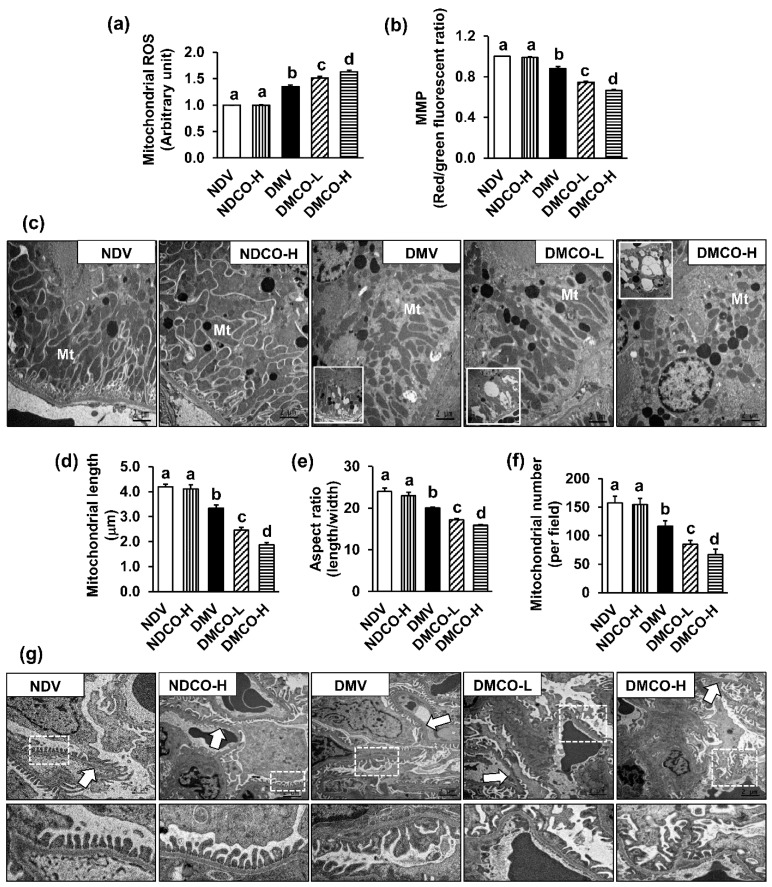
Effects of crocodile oil on renal mitochondrial functions and ultrastructure at the age of 40 weeks: (**a**) mitochondrial reactive oxygen species (ROS) production; (**b**) mitochondrial membrane potential (MMP); (**c**) transmission electron micrographs (original magnification: 3000×) of renal proximal tubules showing mitochondria (Mt) swollen and fragmented with vacuolar changes, plus lysosome formation (inset) in diabetic groups; (**d**–**f**) analysis of mitochondrial morphological changes (mitochondrial length, aspect ratio, and number, respectively); (**g**) transmission electron micrographs (original magnification: 3000×) of glomerulus showing normal thickness and appearance of glomerular basement membrane (arrow) and podocyte foot processes (dot square in upper row with modification in lower row) in nondiabetic groups, whereas thick and wrinkled glomerular basement membranes (arrow) with extensive effacement of podocyte foot processes are presented in the kidney tissues of diabetic rats. Values are mean ± SEM (n = 5 each). NDV: nondiabetic treated with vehicle; NDCO-H: nondiabetic treated with high-dose crocodile oil; DMV: diabetic treated with vehicle; DMCO-L: diabetic treated with low-dose crocodile oil; and DMCO-H: diabetic treated with high-dose crocodile oil. Different lowercase letters denote statistical differences at *p* < 0.05 between groups.

Next, we investigated whether the damaging outcomes of CO on diabetic kidneys could be the consequences of CO disturbing renal mitochondrial function. As expected, a significant increase in mitochondrial ROS production together with a decrease in mitochondrial membrane potential (MMP) were detected in the DMV group compared to the NDV and NDCO-H groups (Figure 4a–b). These changes were dose-dependently intensified in CO-treated diabetic groups. Consistent with mitochondrial function disturbances, electron microscopy of the kidneys from diabetic groups showed fragmented, swollen, and reduced numbers of mitochondria in comparison to the nondiabetic groups (Figure 4c). Additional findings of vacuolar changes and more liposome formation were also observed in diabetes treated with CO. Mitochondrial morphological changes were further confirmed by analysis of mitochondrial length, the aspect ratio of mitochondria (which considers both length and width), and mitochondrial number, which was significantly decreased in all diabetic groups compared to nondiabetic groups (Figure 4d–f). Dose-dependent mitochondrial alterations were evident in CO-treated diabetic groups. At the glomerular site (Figure 4g), diffuse podocyte effacement and glomerular basement membrane thickening were evident in all diabetes cases and were more severe with increased doses of CO. These findings demonstrated that CO exacerbates diabetic kidney injury through both qualitative and quantitative mitochondrial impairment. However, giving CO to nondiabetic rats showed no considerable changes to either mitochondrial function or ultrastructure.

### 3.6. CO Disrupts Signal Transduction Pathway Involved in Mitochondria Homeostasis

**Figure 5:**
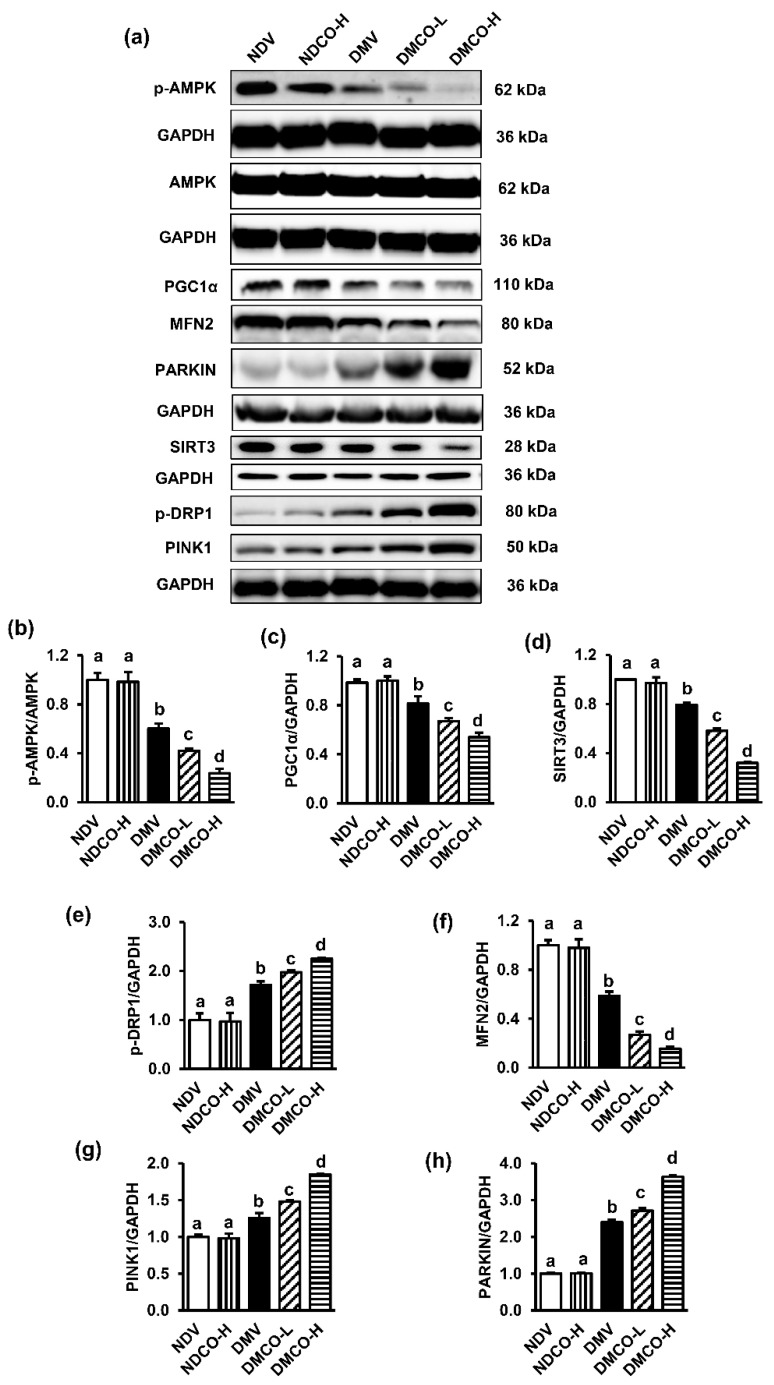
Effects of crocodile oil on the expressions of proteins involved in mitochondrial homeostasis: (**a**) representative images of Western blots; the quantitative analyses of (**b**) p-AMPK/AMPK, (**c**) PGC1α, (**d**) SIRT3, (**e**) pDRP1, (**f**) MFN2, (**g**) PINK, and (**h**) PARKIN. Values are mean ± SEM (n = 3). NDV: nondiabetic treated with vehicle; NDCO-H: nondiabetic treated with high-dose crocodile oil; DMV: diabetic treated with vehicle; DMCO-L: diabetic treated with low-dose crocodile oil; and DMCO-H: diabetic treated with high-dose crocodile oil. Different lowercase letters denote statistical differences at *p* < 0.05 between groups.

The mechanism underlying the unfavorable effects of CO on mitochondria was further explored by evaluating the expressions of signal proteins linked to mitochondrial homeostatic control. The AMPK/PGC1α/SIRT3 axis, which is the key element in controlling several events in the mitochondria, was firstly examined. It was found that CO had no influence on any of these proteins in nondiabetic rats, whereas it downregulated the expressions of p-AMPK/AMPK, PGC1α, and SIRT3 significantly and dose-dependently in diabetic conditions (Figure 5a–d).

Mitochondrial homeostasis cannot be maintained unless mitochondrial biogenesis, dynamics, and mitophagy are physiologically regulated by specific proteins. Hence, the expressions of mitochondrial dynamic proteins (p-DRP1, MFN2) and mitophagy-regulating proteins (PINK1, PARKIN) were further evaluated. Significant increases in p-DRP1 and decreases in MFN2 levels were noticed in all diabetic groups (Figure 5a,e,f), indicating that mitochondrial fission dominated over fusion in diabetes. When diabetic rats were treated with CO, this situation was progressively worsened, more seriously with increasing dose of CO. The mitophagy-regulating proteins PINK1 and PARKIN were also significantly increased in diabetic rats (Figure 5a,g,h). A similar pattern of increasing severity was found in CO-treated diabetic groups when compared with the diabetic control group.

## 4. Discussion

The key challenge of this study is an attempt to turn industrial waste into a valuable product. Crocodile oil (CO) extracted from leftover crocodile adipose tissue was used to explore its effect on the kidney in a rat model of nonobese type 2 diabetes (T2DM). Unfortunately, our results show exacerbation of diabetic kidney injury after long-term CO supplementation through disturbance of mitochondrial homeostasis. The findings, however, bring caution to fatty acid supplementation in diabetes.

Preclinical studies that aim to discover novel therapeutic products against diabetes and to study their possible mechanisms have been using different animal models of genetically or chemically induced T2DM. However, these existing animal models do not show similarity to diabetic patients in the development and progression of diabetic complications. In the present study, spontaneously diabetic Torii (SDT) rats, a recently established animal model of nonobese T2DM, were selected for investigation. SDT rats are an inbred strain of Sprague–Dawley rat that spontaneously develop hyperglycemia, glucose intolerance, and deficient insulin production owing to β-cell degeneration. They also show pancreatic tissue congestion, hemorrhage, inflammation, progressive fibrosis in and around the pancreatic islets, and eventually pancreatic atrophy with advancing age [21,22]. Most importantly, SDT rats demonstrate characteristic features that resembles human diabetic nephropathy in terms of both morphological and functional kidney damage [21,22]. Thus, they are an appropriate model for studying gradual progression of diabetic renal complications.

There are no definite reports about the onset of diabetes in SDT rats, but it varies from 10–20 weeks of age and has an incidence of 100% at 40 weeks [21,22]. In this study, we found that our SDT rats developed insulin resistance at the age of 12 weeks with no renal impairment, as supported by normoglycemia at the non-fasting stage but an impaired glucose tolerance test together with UPCR values similar to those of the nondiabetic control rats at the same age. As we intended to explore whether CO can prevent or slow the development of diabetic kidney injury, we initiated CO supplementation at this age and continued it until the age of 40 weeks, the point at which diabetic renal complications were reported to be fully established [21,22]. Our results demonstrated that all SDT rats exhibited hyperglycemia with hypoinsulinemia along with abnormal pancreatic histomorphology at the end of the study. Azotemia, proteinuria, and abnormalities of kidney morphology were all evident in SDT rats, as observed by increases in blood urea nitrogen and serum creatinine, reduced creatinine clearance, a marked rise in UPCR, and the presence of microalbumin in urine compared with SD rats in the nondiabetic control group. All these characteristics confirmed that the 40-week-old SDT rats in our study suffered from diabetic nephropathy.

T2DM is a multifactorial metabolic disorder. Several factors have implications in the etiology of disease, including dietary influence. Fatty acids are important dietary sources of energy for humans. Further, in conjunction with proteins, fatty acids form important structural components of the cell membrane, mitochondria, and parts of the cytoplasm. These components can regulate membrane structure and function, intracellular signaling pathways, transcription activity, and gene expression [27]. Recently, there have been reports suggesting that intake of dietary fatty acid may play a major role in the prevention and management of T2DM [27]. This leads to an increasing trend of using naturally occurring fatty acids as an adjunct or alternative to the mainstay therapy for diabetes.

Crocodile oil has been reported to contain a variety of fatty acids of benefit to human health. It has also been used in traditional medicine for a variety of diseases [19,20]. Therefore, we assumed that CO may be an alternative to reduce diabetic renal damage. Our results showed that long-term CO supplementation to SDT rats did not decrease metabolic disorders and the damages of pancreatic tissues caused by diabetes. Unexpectedly, CO exacerbated diabetic kidney injury in SDT rats at both functional and structural levels in a dose-dependent manner. These results occurred while supplementation of high-dose CO to nondiabetic rats had no effect on metabolic control or renal structure and function. Based on these findings, it is possible that CO may act on the kidney directly and exert its detrimental effects under diabetic condition independent of glycemic control. This suggestion is supported by a previous publication showing renoprotection by a medicinal plant in SDT rats without lowering blood glucose [28].

Mitochondria play a key role in energy metabolism by producing the high-energy compound ATP and maintaining the membrane potential (Δψm) that is essential to build the proton motive force driving ATP production. Meanwhile, ROS, a byproduct of ATP production by mitochondria, must be eliminated by antioxidants within the mitochondria. Accumulation of ROS due to overproduction under conditions of cellular stress creates the state of mitochondrial oxidative stress that leads to disturbance of mitochondrial homeostasis, mitochondrial dysfunction, and finally, organ damage [4]. Qualitative, quantitative, and functional perturbations in mitochondria have been indicated as contributing to the development and advancement of many diabetic complications, notably diabetic nephropathy [4,29]. As kidneys are one of the most energy-consuming organs in the body, they greatly depend on the integrity of the mitochondria [5,29]. To this end, we firstly examined whether CO supplementation impacted renal mitochondrial function and ultrastructure. Our results revealed that CO had no effect on the mitochondria in nondiabetic rats. However, aggravation of the production of mitochondrial ROS and dissipation of the mitochondrial membrane potential along with destruction of the mitochondrial ultrastructure were evident in CO-supplemented SDT rats, with severity directly proportional to the concentration of CO. The findings suggest that the harmful effects of CO in diabetic kidneys are likely related to mechanisms involved in maintaining homeostasis within the mitochondria.

We further investigated the impact of CO on the key elements of mitochondrial homeostasis. Mitochondrial homeostasis is closely regulated by mitochondrial biogenesis, dynamics, and mitophagy. This machinery acts to maintain mitochondrial structure and function. Its derangement induces mitochondrial damage that is often associated with the induction of mitochondrial ROS production, membrane permeability transition pore (mPTP) opening, and dissipation of membrane potential [29,30]. As expected, CO potentiated the downregulation of pAMPK/AMPK, PGC1α, and SIRT3 expressions in diabetes. These signaling proteins are essential in mitochondrial biogenesis, which is an integral part of maintaining mitochondrial quality. PGC1α and SIRT3 also play a role in controlling a multitude of processes within the mitochondria, including respiratory chain activity, redox balance, and cell death [24,29,30]. Further, CO supplementation with diabetes worsened the process of mitochondrial dynamics, as shown by the increased mitochondrial fission proteins (pDRP1) and decreased mitochondrial fusion proteins (MFN2), causing mitochondrial fragmentation, release of apoptogenic factors, and more phagolysosome formation, as observed in our electron micrograph. Fission and fusion of mitochondria are the major adaptive mechanisms in ATP production, and, therefore, the balance between them can control cellular metabolism, linking mitochondrial structure to function [30]. Several preclinical studies of diabetic nephropathy have also shown that increased fission is associated with markers of renal damage, such as changes in creatinine clearance, impaired excretory functions, and proteinuria [31,32]. Regarding mitophagy, the damaged mitochondria are normally digested by the enzymes from autophagosome formation. Mitochondrial fragmentation has been reported to facilitate mitophagy. Accumulation of autophagosomes containing mitochondria has been found in human [33] and rodent diabetic kidneys [34]. Consistent with our study, we also detected increased expressions of PINK and PARKIN in SDT rats, which was amplified following CO supplementation. Collectively, our results provide evidence to demonstrate the detrimental effects of CO in exacerbating diabetic kidney injury, which is most likely caused by disruption of mitochondrial homeostasis.

In this study, we introduced CO as a potential therapeutic supplement for T2DM to prevent or reduce diabetic renal injury. Surprisingly, the results turned out to be in the opposite direction. Previous toxicological studies in Wistar rats using CO extract from the same species as in our study did not show any signs of toxic effects, moribund, or mortality up to 2000 mg/kg BW [35]. Our results in nondiabetic SD rats are consistent with this report, while the negative effects of CO shown in our study were limited only to diabetic rats. It is possible that certain elements in CO may influence pre-existing metabolic disorders in diabetes.

We further analyzed fatty acid constituents in CO and found that nearly 50% are MUFAs, with oleic acid accounting for almost 41%. Oleic acid is one of the MUFA omega-9 fatty acids. Several studies have revealed the advantages of oleic acid in T2DM through its insulin-secreting, anti-inflammatory, anti-hyperlipidemic, and antioxidant actions [36,37,38]. In our study, CO did not worsen diabetes-induced metabolic disorders, which may be due to the protective benefits of oleic acid. Moreover, adverse effects of oleic acid have also been reported. One study suggested oleic acid stimulates transcription of p66shc, leading to increased mitochondrial ROS production and, consequently, mitochondrial depolarization and associated cell injury [39]. This could possibly result in the worsening of diabetic kidney injury by CO as observed in our study. As the advantages and disadvantages of oleic acid in diabetes and diabetic kidney disease remain controversial. The available information is insufficient for us to draw any definite conclusions regarding the impacts of oleic acid in our present study.

PUFAs can be categorized into omega-3 and omega-6 fatty acids. Linoleic acids and α-linoleic acids are essential fatty acids that must be obtained from the diet [12]. It is believed that daily PUFA supplementation can help improve diabetes, as several reports have demonstrated that plant- or animal-derived omega-3 and omega-6 PUFAs attenuate hypertension, inflammation, glomerulosclerosis, and albuminuria in most experimental studies of diabetic nephropathy [40]. Omega-3 fatty acid supplementation has also been shown to be associated with a significantly reduced risk and delayed progression of end-stage renal disease (ESRD) [41]. Clinical trials in diabetes patients with omega-3 PUFAs, especially DHA and EPA, have reported that hypoglycemic and antidiabetic effects of omega-3 PUFAs are derived from insulin-sensitizing actions of DHA and EPA via SREBP, PPAR, and GLP-1 production [42]. However, there are some limitations and variations to these studies, and thus, the obtained results are still debated [43].

Omega-6 and omega-3 work together to promote health, but they must be balanced in the diet. The best ratio of omega-6 to omega-3 ranges from 1:1 to 4:1, and Western diets contain a high content of omega-6 and a very high omega-6/omega-3 ratio (15:1–16.7:1), contributing to the rising rate of diabetes and inflammatory disorders in Western countries [40,44]. Higher ratios promote the development of diabetes and its complications by a process of metabolic imbalance that takes part in pro-inflammatory, pro-thrombotic, pro-aggregatory potentials. High intake of omega-6 produces eicosa- metabolic products from arachidonic acids, especially prostaglandins, thromboxanes, leukotrienes, and lipoxins in a large quantity. They induce inflammation, large amount of ROS production, and oxidative stress [40]. In our study, CO is composed of omega-3 fatty acids (1.42%) and omega-6 fatty acids (23.70%), causing the ratio of omega-6/omega-3 to be 16.7/1. Accordingly, it is suggested that this high ratio of omega-6/omega-3 in CO may contribute to the unfavorable effects observed in our diabetic rats.

Apart from PUFA, CO also contains a similar amount of saturated fatty acids (SFA). Palmitic acid, one of the long-chained saturated fatty acids (LCSFA), provides increased levels of plasma non-esterified fatty acid NEFA. Increased NEFA levels lead to oversupply of lipids into the mitochondria through FA translocase or CD36. This directs incomplete FA oxidation and then increases generation of ROS, with concomitant mitochondrial stress [45,46]. In the present study, palmitic acid contributes the major component of SFA in CO, thus it may lead to increased mitochondrial ROS production, change in mitochondrial membrane potential, and mitochondrial stress, especially in renal tissues that have been primed by glycemic stress.

## 5. Conclusions

To summarize, the present study provides new insight to demonstrate an aggravation of CO on diabetic nephropathy through the disruption of mitochondrial homeostasis, causing mitochondrial dysfunction. Based on our findings and the existing literature, it is proposed that the deleterious effects of CO are likely due to its SFA palmitic acid component and, particularly, the improper ratio of omega-6/omega-3. To our knowledge, this is the first report about the effects of CO in T2DM and renal complications. Since the study used a model of type 2 diabetes that was very similar to that in humans. These findings are likely to be applicable to real life in terms of raising awareness of fatty acid supplementation in diabetic patients with renal dysfunction.

## Figures and Tables

**Table 1:** Fatty acid compositions of CO.

Fatty Acid Compositions	Quantity
**Saturated fatty acids (SFAs)**	**28.63 ± 0.08**
Palmitic acid (C16:0)	21.98 ± 0.75
Stearic acid (C18:0)	5.35 ± 0.21
**Monounsaturated fatty acids (MUFAs)**	**46.02 ± 1.79**
Palmitoleic acid (C16:1n7)	4.43 ± 0.27
Oleic acid (C18:1n9c)	40.87 ± 1.62
**Polyunsaturated fatty acids (PUFAs)**	**25.35 ± 1.74**
Linoleic acid, LA (C18:2n6)	23.29 ± 1.56
α-linolenic acid, ALA (C18:3n3)	1.05 ± 0.16
Eicosapentenoic acid, EPA (C20:5n3)	0.04 ± 0.00
Docosahexaenoic acid, DHA (C22:6n3)	0.17 ± 0.01

Values are mean ± SEM (n = 3) and expressed in g/100 g final crocodile oil product.

**Table 2:** Metabolic and renal function parameters in all experimental groups at the age of 8 and 12 weeks.

	Age 8 Weeks		Age 12 Weeks	
	ND	SDT	ND	SDT
**Metabolic parameters**				
Body weight (g)	207.00 ± 1.53 ^a^	199.67 ± 3.22 ^a^	491.50 ± 5.43 ^b^	394.33 ± 8.81 ^b^
Food intake (g/day)	23.60 ± 0.36 ^a^	23.10 ± 0.27 ^a^	28.70 ± 0.56 ^b^	25.73 ± 0.61 ^b^
Non-fasting plasma glucose (mg/dL)	124.70 ± 1.16 ^a^	126.67 ± 1.03 ^a^	144.60 ± 2.57 ^b^	149.27 ± 2.26 ^b^
**Oral glucose tolerance test (OGTT)**				
Fasting plasma glucose (mg/dL)	-	-	124.63 ± 5.56 ^a^	102.97 ± 9.96 ^a^
Fasting plasma insulin (ng/mL)	-	-	0.24 ± 0.00 ^a^	1.38 ± 0.09 ^b^
TAUC (mg/dL/h)	-	-	234.26 ± 15.01 ^a^	459.10 ± 40.71 ^b^
**Renal function parameters**				
Serum creatinine (mg/dL)	0.45 ± 0.02 ^a^	0.46 ± 0.01 ^a^	0.46 ± 0.01 ^a^	0.45 ± 0.01 ^a^
Urine protein-to-creatinine ratio (UPCR)	0.06 ± 0.01 ^a^	0.06 ± 0.01 ^a^	0.07 ± 0.01 ^a^	0.08 ± 0.01 ^a^

Values are mean ± SEM. ND: nondiabetic Sprague–Dawley rats (n = 10); SDT: spontaneously diabetic Torii rats (n = 15); TAUC: total area under the curve. Different lowercase letters denote statistical differences at *p* < 0.05 between groups.

**Table 3:** Metabolic parameters in all experimental groups at the age of 40 weeks.

	NDV	NDCO-H	DMV	DMCO-L	DMCO-H
Bodyweight (g)	760.50 ± 27.64 ^a^	788.75 ± 22.16 ^a^	352.50 ± 3.35 ^b^	353.75 ± 11.77 ^b^	345.00 ± 6.71 ^b^
Food intake (g/day)	26.50 ±1.12 ^a^	29.00 ± 1.22 ^a^	53.50 ± 1.60 ^b^	52.00 ± 0.77 ^b^	53.00 ± 3.13 ^b^
Non-fasting plasma glucose (mg/dL)	120.12 ± 5.74 ^a^	117.67 ± 4.17 ^a^	483.89 ± 5.10 ^b^	487.63 ± 3.39 ^b^	486.74 ± 29.81 ^b^
Plasma insulin (ng/mL)	1.09 ± 0.05 ^a^	0.73 ± 0.13 ^a^	0.06 ± 0.01 ^b^	0.06 ± 0.01 ^b^	0.09 ± 0.02 ^b^

Values are mean ± SEM (n = 5 each). NDV: nondiabetic rats treated with vehicle; NDCO-H: nondiabetic rats treated with high-dose crocodile oil; DMV: diabetic rats treated with vehicle; DMCO-L: diabetic rats treated with low-dose crocodile oil; and DMCO-H: diabetic treated with high-dose crocodile oil. Different lowercase letters denote statistical differences at *p* < 0.05 between groups.

## Data Availability

The data supporting reported results are available on request from the corresponding author. A sample of the crocodile oil is available from the authors.
